# Dog, cat, bird, fish, and other pet ownership and mortality: Evidence from the HILDA cohort

**DOI:** 10.1371/journal.pone.0305546

**Published:** 2024-08-14

**Authors:** Yu Taniguchi, Tomoko Ikeuchi, Jongsay Yong

**Affiliations:** 1 Japan Environment and Children’s Study Programme Office, National Institute for Environmental Studies, Tsukuba, Japan; 2 Melbourne Institute of Applied Economic and Social Research, The University of Melbourne, Melbourne, Australia, Faculty of Business and Economics Building 111 Barry Street, Melbourne, VIC, Australia; 3 Research Team for Social Participation and Healthy Aging, Tokyo Metropolitan Institute for Geriatrics and Gerontology (TMIG), Tokyo, Japan; 4 Research Team for Human Care, Tokyo Metropolitan Institute for Geriatrics and Gerontology (TMIG), Tokyo, Japan; UFSJ: Universidade Federal de Sao Joao del-Rei, BRAZIL

## Abstract

This study used the nationally representative prospective study of the Household, Income and Labour Dynamics in Australia (HILDA) survey cohort to examine the association of pet ownership (dog, cat, bird, fish, and others) with the risk of all-cause mortality using propensity score matching based on a wide range of factors. The study sample included 15,735 participants who completed the questionnaire on pet ownership in 2018. The HILDA survey sample was matched to the National Death Index through 2022 to assess death during the follow-up period. Statistical analysis was weighted by the inverse of the propensity score in the generalized estimating equation. During the 4-year follow-up period, 377 of 15,735 (2.4%) participants died. The odds ratios (ORs) for all-cause mortality were 0.77 (95%CI: 0.59–0.99) for dog owners compared to non-pet owners after controlling for related socio-demographic, physical, psychological, and social factors. The Sobel test showed a partial mediating effect of physical activity level on the relationship between dog ownership and all-cause mortality. Ownership of cats, birds, fish, and others showed no clear association with mortality, despite owners having similar socio-demographics characteristics to dog owners. Companionship and exercise of a pet dog may be recommended as a component of health promotion policy, and may have an important role to play in promoting health aging.

## 1. Introduction

Studies over the last few decades on human–animal interaction have reported physical, psychological, and social effects of pets on their owners and those they interact with [1]. Accumulated evidence primarily relates to physical effects, and shows that pet ownership may have positive effects on physical activity levels [2]; blood pressure response [3]; activities of daily living levels [4]; and cardiovascular [5], frailty [6, 7], dementia [8] and mortality risks [9, 10].

Most previous studies of human–animal interaction have focused on dog and/or cat ownership and health outcomes. In particular, dog owners have been shown to have lower risk of death, which is attributed to reduced risk of cardiovascular death [11–13]. A systematic review and meta-analysis of 10 studies published between 1950 and 2019 concluded that dog ownership is associated with lower risk of death over the long term, possibly driven by a reduction in cardiovascular mortality [14]. Meanwhile, a longitudinal study of 13,929 adults in the United States reported that dog owners had slightly lower risk of dying from colorectal cancer [15]. Our previous study of 11,233 older adults in Japan revealed that dog ownership appears to protect against incident disability and all-cause mortality, and that older dog owners with regular exercise, including dog walking, had the lowest odds ratio for the onset of disability [9]. Thus, dog ownership might be associated with reduced risk of death through a decrease in cardiovascular disease (CVD) events via regular exercise.

Cat ownership is also suggested to be effective in reducing mortality risk. A longitudinal study of 3,964 adults in the United States reported that owning a cat rather than a dog was significantly associated with reduced hazard of dying from CVD events [16]. Several studies have reported positive associations between pet ownership, including cat ownership, and mortality risk [10, 17], albeit that negative effects of cat ownership have also been raised [15, 18–20].

A few studies have examined the association between pets other than dogs and cats and mortality risk. One study using data from NHANES III showed that bird ownership was associated with increased risk of dying from cancer, especially in women [19]. Other studies from NHANES III also focused on bird ownership, in addition to dog and cat ownership, and reported non-significant associations with mortality [15, 18]. These studies collected data for fish, rodent, rabbit, reptile, and farm pet ownership, but did not examine associations with mortality due to the relatively small number of owners.

Several clinical trials have shown the positive effects of an attachment to companion animals. An animal-assisted intervention with horses had a positive effect on physical and psychological function in dementia patients [21]. Watching a videotape of an aquarium had a substantial impact on physiological stress [22], and petting an animal such as a rabbit or turtle reduced state-anxiety [23]. These findings may suggest that ownership of pets such as horses, fish, and rabbits have positive effects on mortality; nevertheless, evidence to confirm this notion is insufficient.

This study reports a prospective study of the HILDA cohort, a nationally representative longitudinal sample of Australian households. The study have two objectives: (1) to identify socio-demographic, physical, psychological, and social factors associated with pet ownership (i.e., dogs, cats, birds, fish, horses, rabbits, and others) and (2) to examine these respective associations with risk of all-cause mortality, using propensity score matching based on a wide range of factors related to ownership. Implementation of a propensity score matching design overcomes the difficulty of examining the direct health effects of pet ownership, and instead examine the effects of each pet ownership on the risk of mortality, net of baseline health.

## 2. Methods

### 2.1. Study population

The Household, Income and Labour Dynamics in Australia (HILDA) survey is Australia’s first and only large-scale, nationally representative household panel survey. The design of the HILDA survey has been published elsewhere [24–26]. Briefly, its primary objective is to support research questions falling within three broad and inter-related areas of income, labour market and family dynamics. The HILDA Survey is a household-based panel study of Australian households which interviews all household members (15 years and over) of selected households and then re-interviews the same people in subsequent years. The initial sample was selected using a multistage sampling approach. First, a probability proportional to size sampling technique was initiated to select 488 Census Collection Districts (CDs), which are the smallest spatial unit used for collecting and recording Census data by the Australian Bureau of Statistics. Each CD covered approximately 200–250 households. Second, a sample of 22–34 dwellings was randomly selected from each of the CDs. Finally, a maximum of three households from each dwelling was selected. This process resulted in a total of 12,252 households in 2001. In 2011 a Top-Up sample of 2,153 households was added to allow better representation of the Australian population using the same methodology as the original sample [27].

The survey covers a range of dimensions, including social, demographic, health and economic conditions. Data were collected using both face-to-face interviews with trained interviewers and self-completion questionnaires. The dataset used in the present study is the 22nd release of HILDA data, incorporating data collected from 2001 through 2022 (https://dataverse.ada.edu.au/dataverse/DSSLongitudinalStudies).

### 2.2. Definition of pet ownership

Participants were asked if they had any pet in 2018 (Wave 18). Those with current pet ownership were asked to indicate the pet species, i.e., dog, cat, bird, fish, horse, or other. If participants selected other, they were asked to specify which type. From the description, rabbit, guinea pig/hamster, lizard, snake, turtle/tortoise, sheep, cow, goat, rat/mouse, alpaca, and others were identified. In this study, we used ownership of dogs, cats, birds, fish, and others (horse, rabbit, guinea pig/hamster, lizard, snake, turtle/tortoise, sheep, cow, goat, rat/mouse, alpaca, and other), because proportions of these ownerships exceeded 5% of the sampled households for each.

### 2.3. Socio-demographic, physical, psychological, social variables and mortality

Socio-demographic variables included sex (male, female), age, marital status (legally married, de facto, separated, divorced, widowed, never married and not de facto), family member (non-family member, member of first family in household, member of second family in household, member of third family in household), residential area (major city, inner regional, outer regional, remote, very remote), housing (owner/currently paying off home, renting, involved in a rent-buy scheme, living rent-free/life tenure), income [28, 29], per-person fees paid to health practitioners, and per-person use of medicines, prescription drugs, pharmaceuticals, and alternative medicines in 2018 (Wave 18).

Physical variables included long-term impairment or disability (yes, no), limitation in moderate and vigorous activities (limited a lot, limited a little, not limited at all), walking more than one kilometer (limited a lot, limited a little, not limited at all), and bathing or dressing oneself (limited a lot, limited a little, not limited at all) in 2018 (Wave 18). Furthermore, data on minutes per week of moderate physical activity, vigorous physical activity [30], total physical activity of metabolic equivalents (MET) [30] were used, in addition to the number of doctor visits, and diagnosis with a serious illness (yes, no), asthma, anxiety, arthritis or osteoporosis, any type of cancer, chronic bronchitis or emphysema, type 2 diabetes, depression, high blood pressure or hypertension, heart disease, or other mental illness in 2017 (Wave 17) (these variables were not collected in Wave 18).

Psychological variables included self-assessed health (excellent, very good, good, fair, poor), SF-36 mental health [31], frequent feeling of deep loneliness, satisfaction with the residential home, feeling of happiness as a person; feeling calm and peaceful; feeling of being a nervous person; feeling down; feeling full of life; and having a lot of energy (all of the time, most of the time, a good bit of the time, some of the time, a little of the time, none of the time) in 2018 (Wave 18).

Social variables included participation in physical activity (not at all, less than once a week, 1 to 2 times a week, 3 times a week, more than 3 times a week, every day); neighbors help each other (never happens, very rare, not common, fairly common, very common); neighborhood is close-knit; chatting with neighbors (never, rarely, occasionally, sometimes, often, very often); neighborhood can be trusted; most people can be trusted; and social gatherings with friends/relatives in 2018 (Wave 18).

The HILDA Survey sample was matched to the National Death Index (NDI), that is a database developed and maintained by the Australian Institute of Health and Welfare. The database is a listing of all deaths that have occurred in Australia since 1980. The data from 2001 through 2022 were covered in Wave 22 [32]. In this study, we defined the incidence of death by the variable age at death, and used the variable year of death to calculate the follow-up period. Participants without a death indicator by 2022 were treated as censored.

### 2.4. Eligibility criteria

To be eligible for the study, individuals had to complete the questionnaire on pet ownership in 2018 (Wave 18). A total of 15,735 participants were included in this study. For variables with missing values defined in the 22nd release of the HILDA data, missing values (-1 or less) were used as is, and variables with undefined missing values were treated as blanks.

This study used unit record data from HILDA conducted by the Australian Government Department of Social Services (DSS). However, the findings and views reported in this paper are those of the authors and should not be attributed to the Australian Government, DSS, or any DSS contractors or partners. This study did not require ethical approval as the analysis used de-identified data only, existing as unit record data from the HILDA Survey. Nevertheless, the authors completed and signed the Confidentiality Deed Poll and sent it to NCLD (ncldresearch@dss.gov.au) and ADA (ada@anu.edu.au) before the data applications were approved. The datasets analyzed and/or generated during the current study are accordingly subject to this signed confidentiality deed.

### 2.5. Statistical analysis

First, relationships between socio-demographic, physical, psychological, and social factors and pet ownership were tested using the chi-square test or t-test. Pet ownership was analyzed separately for dog, cat, bird, fish, and others ownership. Second, associations between pet ownership and all-cause mortality were estimated using an inverse probability of treatment weighted logistic regression model with propensity score. Standard error of the estimated value was calculated with robust variance. Weights were calculated based on the results of the relationship between socio-demographic, physical, psychological, and social factors with each pet ownership. The application of these weights to the study population creates a pseudopopulation in which confounders are equally distributed across exposed and unexposed groups. We determined for Model 1 to include socio-demographic factors and Model 2 to include socio-demographic, physical, psychological, and social factors as explanatory variables after discussion among the all authors. The weightages or coefficients were determined by the regression algorithm. Model 1 included sex, age, marital status, family members, housing, and income. Model 2 added limitation in vigorous activities and in walking more than one kilometer, self-assessed health, SF-36 mental health, neighbors help each other out, social gatherings with friends/relatives with friends/relatives, any type of cancer, type 2 diabetes, and high blood pressure or hypertension in Model 1. Using an inverse probability of treatment weighted logistic regression model with propensity score, we conducted a sensitivity analysis without death until the first year of the follow-up period. As a supplementary analysis, we used mediation analysis of physical activity level (total physical activity MET) on the relationship between pet ownership and all-cause mortality, with physical activity level used as a mediator variable based on previous evidence [8, 9] to explore the underlying mechanism between pet ownership and mortality. Statistical analyses were conducted using Stata SE (version 18; Stata Corp, College Station, TX, USA) and SPSS (version 23; IBM Corp, Armonk, NY, USA).

## 3. Results

### 3.1. Sample characteristics

Data from the baseline survey of 15,735 participants showed that the mean (SD) age of participants was 46.1 (19.1) years (minimum 15, maximum 99 years), and that 53.1% were women. 47.3% were legally married and 16.5% had a de facto status; 81.8% were members of the first family in the household; 61.8% lived in a major city and 26.2% lived in inner regional Australia; 67.9% owned or were currently paying off a mortgaged house and 29.3% were renters or paid board; average income was A$102,610 (74,888); average per-person fees paid to health practitioners was A$911 (2,112); and the average per-person payments for medicines, prescriptions, pharmaceuticals, and alternative medicines was A$434 (732). 9,525 of 15,735 (60.5%) were pet owners and the remaining 6,210 (39.5%) were non-pet owners. Among the 9,525 pet owners, 6,898 (43.8%) owned dogs, 3,717 (23.6%) owned cats, 1,532 (9.7%) owned birds, 1,203 (7.7%) owns fish, and 1,028 (6.5%) owned other pets.

### 3.2. Related socio-demographic, physical, psychological, and social factors for pet ownership

Compared to non-pet owners, pet owners were more likely to be female (54.7%), younger (mean = 43.4), married (48.5%), a member of the first family in the household (7.9%), living in one’s own/currently paying off a house (71.0%), have higher income (mean = 109,440), have no limitation in moderate (75.3%) or vigorous activities (44.4%), no limitation in walking more than one kilometer (76.7%), no limitation in bathing or dressing (90.4%), have higher moderate (mean = 171.1), vigorous (mean = 114.0), and total physical activity (mean = 2393), have asthma (26.0%), have anxiety (27.7%), depression (28.3%), and other mental illness (5.8%), feel that they are a nervous person (6.0%), feel down (5.3%), participate in physical activity (11.1%), have neighbors who help each other (19.1%), and chat with neighbors (45.2%). Pet owners were also less likely to live in a major city (56.8%) and less likely to have long-term impairment or disability (43.0%), a diagnosis of arthritis or osteoporosis (32.5%), any type of cancer (5.4%), chronic bronchitis (4.0%) or emphysema (4.0%), type 2 diabetes (9.5%), high blood pressure or hypertension (34.2%), or heart disease (7.3%). They also had a lower SF-36 score (mean = 71.5), were less likely to be happy (54.4%), were less likely to feel calm and peaceful (38.3%), had a lower close-knit neighborhood score (mean = 3.94), were less likely to trust their neighborhood (mean = 4.71) and people in general (mean = 4.80), and were less likely to socialize with friends/relatives (21.2%) (Table 1). Socio-demographic characteristics such as more likely to be female, younger age, married, and member of the first family in the household were common among all pet owners (Tables 1 and 2).

**Table 1:** Relationship between socio-demographic, physical, psychological, and social factors and pet ownership (any pet, dog, and cat ownership).

Variable	Any pet			Dog			Cat		
	No (n = 6,210)	Yes (n = 9,525)	P-value	No (n = 8,837)	Yes (n = 6,898)	P-value	No (n = 12,018)	Yes (n = 3,717)	P-value
**SOCIO-DEMOGRAPHICS**									
** Sex (% female)**	**50.6%**	**54.7%**	**P<0.001**	**52.2%**	**54.2%**	**P<0.001**	**51.9%**	**57.0%**	**P<0.001**
** Age**			**P<0.001**			**P<0.001**			**P<0.001**
mean	**50.2**	**43.4**		**48.7**	**42.6**		**47.0**	**43.1**	
SE	**0.3**	**0.2**		**0.2**	**0.2**		**0.2**	**0.3**	
** Marital status (%)**			**P<0.001**			**P<0.001**			**P<0.001**
Legally married	**45.6%**	**48.5%**		**45.6%**	**49.5%**		**48.1%**	**44.7%**	
De facto	**13.3%**	**18.6%**		**14.7%**	**18.8%**		**15.7%**	**19.3%**	
Separated, divorced, widowed, never married and not de facto	**41.1%**	**32.9%**		**39.6%**	**31.8%**		**36.2%**	**36.0%**	
** Family member (non-family member %)**	**26.5%**	**10.6%**	**P<0.001**	**22.9%**	**9.1%**	**P<0.001**	**18.7%**	**10.9%**	**P<0.001**
** Residential area (%)**			**P<0.001**			**P<0.001**			**P<0.001**
Major city	**69.4%**	**56.8%**		**67.9%**	**54.0%**		**63.8%**	**55.3%**	
Inner regional, outer regional, remote, very remote	**36.2%**	**44.7%**		**32.1%**	**46.0%**		**36.2%**	**44.7%**	
** Housing (%)**			**P<0.001**			**P<0.001**			**P<0.001**
Owner/currently paying off home	**63.3%**	**71.0%**		**64.1%**	**72.8%**		**68.2%**	**67.1%**	
Renting (or paying board), involved in a rent-buy scheme or Living rent-free/Life Tenure	**36.7%**	**29.0%**		**35.9%**	**27.2%**		**31.8%**	**32.9%**	
** Income(A$)**			**P<0.001**			**P<0.001**			**P<0.001**
mean	92,135	109,440		95,265	112,020		**101,787**	**105,271**	
SE	942	764		801	882		**675**	**1,272**	
** Annual household expenditure–per person level–fees paid to health practitioners (A$)**			P = 0.122			P = 0.152			**P = 0.039**
mean	948	885		936	877		**934**	**834**	
SE	33	25		26	31		**24**	**35**	
** Annual household expenditure–per person level–medicines, prescriptions, pharmaceuticals, alternative medicines (A$)**			P = 0.329			P = 0.712			P = 0.585
mean	426	440		436	431		436	427	
SE	10	10		9	11		8	15	
**PHYSICAL VARIABLES**									
** Long-term impairment or disability (Yes %)**	**48.1%**	**43.0%**	**P<0.001**	**31.0%**	**27.4%**	**P<0.001**	**28.9%**	**31.1%**	**P = 0.032**
** Limitation in activities: moderate activities (Not limited at all %)**	**68.8%**	**75.3%**	**P<0.001**	**70.3%**	**75.9%**	**P<0.001**	72.4%	73.8%	P = 0.369
** Limitation in activities: vigorous activities (Not limited at all %)**	**38.2%**	**44.4%**	**P<0.001**	**38.9%**	**45.8%**	**P<0.001**	**41.7%**	**42.6%**	**P = 0.041**
** Walking more than one kilometer (Not limited at all %)**	**71.6%**	**76.7%**	**P<0.001**	**72.7%**	**77.3%**	**P<0.001**	74.7%	74.8%	P = 0.816
** Bathing or dressing yourself (Not limited at all %)**	**87.5%**	**90.4%**	**P<0.001**	**88.2%**	**90.6%**	**P<0.001**	89.2%	89.6%	P = 0.827
** Physical Activity in Wave 17**									
** Moderate physical activity (Minutes per week)**			**P<0.001**			**P<0.001**			P = 0.052
mean	**142.4**	**171.1**		**147.6**	**175.5**		157.5	167.4	
SE	**3.2**	**2.9**		**2.8**	**3.5**		2.5	4.6	
** Vigorous physical activity (Minutes per week)**			**P = 0.014**			**P<0.001**			P = 0.518
mean	**90.8**	**114.0**		**92.8**	**120.3**		104.3	106.8	
SE	**2.5**	**2.3**		**2.1**	**2.8**		1.9	3.6	
** Total physical activity MET (Minutes per week)**			**P<0.001**			**P<0.001**			P = 0.289
mean	**2028**	**2393**		**2071**	**2478**		2236	2291	
SE	**32**	**30**		**27**	**36**		25	47	
** Number of doctor visits in Wave 17**			P = 0.205			P = 0.067			P = 0.283
mean	5.0	4.8		5.0	4.8		4.8	5.0	
SE	0.1	0.1		0.1	0.1		0.1	0.1	
** Diagnosed with a serious illness in Wave 17**									
** Asthma (Yes %)**	**19.8%**	**26.0%**	**P<0.001**	**21.0%**	**27.0%**	**P<0.001**	**22.8%**	**25.5%**	**P<0.001**
** Anxiety (Yes %)**	**18.2%**	**27.7%**	**P<0.001**	**20.6%**	**28.3%**	**P<0.001**	**21.5%**	**30.9%**	**P<0.001**
** Arthritis or osteoporosis (Yes %)**	**41.6%**	**32.5%**	**P<0.001**	**38.8%**	**32.6%**	**P<0.001**	**37.8%**	**31.5%**	**P<0.001**
** Any type of cancer (Yes %)**	**8.2%**	**5.4%**	**P<0.001**	**7.3%**	**5.4%**	**P<0.001**	**6.9%**	**5.2%**	**P<0.001**
** Chronic bronchitis or emphysema (Yes %)**	**4.4%**	**4.0%**	**P<0.001**	**4.3%**	**3.9%**	**P<0.001**	**4.2%**	**4.0%**	**P<0.001**
** Type 2 diabetes (Yes %)**	**12.1%**	**9.5%**	**P<0.001**	**11.8%**	**8.8%**	**P<0.001**	**10.7%**	**10.2%**	**P<0.001**
** Depression (Yes %)**	**18.4%**	**28.3%**	**P<0.001**	**21.1%**	**28.6%**	**P<0.001**	**21.9%**	**31.5%**	**P<0.001**
** High blood pressure or hypertension (Yes %)**	**43.7%**	**34.2%**	**P<0.001**	**41.3%**	**33.6%**	**P<0.001**	**40.3%**	**31.4%**	**P<0.001**
** Heart disease (Yes %)**	**12.6%**	**7.3%**	**P<0.001**	**11.1%**	**7.2%**	**P<0.001**	**10.3%**	**7.0%**	**P<0.001**
** Other mental illness (Yes %)**	**4.2%**	**5.8%**	**P<0.001**	**4.7%**	**5.7%**	**P<0.001**	**4.6%**	**6.6%**	**P<0.001**
**PSYCHOLOGICAL VARIABLES**									
** Self-assessed health (Excellent, very good %)**	44.3%	45.5%	P = 0.424	**44.2%**	**46.1%**	**P = 0.049**	**45.9%**	**42.1%**	**P = 0.001**
** SF-36 mental health**			**P<0.001**			**P<0.001**			**P<0.001**
mean	**73.3**	**71.5**		**72.7**	**71.6**		**72.9**	**70.0**	
SE	**0.2**	**0.2**		**0.2**	**0.2**		**0.2**	**0.3**	
** Often feel very lonely**			P = 0.093			P = 0.757			**P<0.001**
mean	2.72	2.77		2.75	2.76		**2.72**	**2.86**	
SEE	0.02	0.02		0.02	0.02		**0.02**	**0.03**	
** Satisfaction with the residential home**			P = 0.320			P = 0.126			**P<0.001**
mean	7.97	7.95		7.94	7.98		**7.98**	**7.87**	
SE	0.02	0.01		0.02	0.02		**0.01**	**0.02**	
** Feel happiness as a person (%)**			**P<0.001**			P = 0.065			**P<0.001**
All of the time, most of the time	**59.3%**	**54.4%**		57.3%	55.2%		**58.0%**	**51.1%**	
A good bit of the time, some of the time, a little of the time, none of the time	**40.7%**	**45.6%**		42.7%	44.8%		**42.0%**	**48.9%**	
** Feel calm and peaceful (%)**			**P<0.001**			**P = 0.001**			**P<0.001**
All of the time, most of the time	**43.5%**	**38.3%**		**41.8%**	**38.5%**		**41.9%**	**35.4%**	
A good bit of the time, some of the time, a little of the time, none of the time	**56.5%**	**61.7%**		**58.2%**	**61.5%**		**58.1%**	**64.6%**	
** Feel I am a nervous person (%)**			**P = 0.001**			P = 0.084			**P<0.001**
All of the time, most of the time	**4.6%**	**6.0%**		5.1%	5.8%		**5.0%**	**6.8%**	
A good bit of the time, some of the time, a little of the time, none of the time	**95.4%**	**94.0%**		94.9%	94.2%		**95.0%**	**93.2%**	
** Feel down (%)**			**P<0.001**			**P = 0.016**			**P<0.001**
All of the time, most of the time	**4.3%**	**5.3%**		**4.6%**	**5.3%**		**4.5%**	**6.5%**	
A good bit of the time, some of the time, a little of the time, none of the time	**95.7%**	**94.7%**		**95.4%**	**94.7%**		**95.5%**	**93.5%**	
** Feel full of life (%)**			P = 0.067			P = 0.084			**P<0.001**
All of the time, most of the time	43.2%	41.6%		41.7%	42.9%		**43.7%**	**37.7%**	
A good bit of the time, some of the time, a little of the time, none of the time	56.8%	58.4%		58.3%	57.1%		**56.3%**	**62.3%**	
** Have a lot of energy (%)**			P = 0.054			**P = 0.001**			**P<0.001**
All of the time, most of the time	31.4%	29.5%		**30.4%**	**30.0%**		**31.4%**	**26.3%**	
A good bit of the time, some of the time, a little of the time, none of the time	68.6%	70.5%		**69.6%**	**70.0%**		**68.6%**	**73.7%**	
**SOCIAL VARIABLES**									
** Participate in physical activity (%)**			**P<0.001**			**P<0.001**			**P = 0.024**
Not at all, less than once a week, 1 to 2 times a week or 3 times a week	**91.9%**	**88.9%**		**60.2%**	**61.3%**		**60.6%**	**60.8%**	
More than 3 times a week or Every day	**8.1%**	**11.1%**		**39.8%**	**38.7%**		**39.4%**	**39.2%**	
** Neighborhood: Neighbors help each other out (%)**			**P<0.001**			**P<0.001**			P = 0.602
Never happens, very rare	**19.9%**	**18.0%**		**19.4%**	**19.0%**		18.7%	19.0%	
Not common, fairly common	**61.9%**	**62.8%**		**62.4%**	**60.2%**		62.4%	62.5%	
Very common	**18.2%**	**19.1%**		**18.2%**	**20.7%**		18.9%	18.5%	
** Close-knit neighborhood**			**P = 0.025**			P = 0.968			**P<0.001**
mean	**4.00**	**3.94**		3.96	3.97		**4.00**	**3.86**	
SE	**0.02**	**0.02**		0.02	0.02		**0.01**	**0.03**	
** Chat with neighbors (%)**			**P = 0.026**			P = 0.186			**P<0.001**
Never, rarely	**32.1%**	**32.5%**		32.6%	32.1%		**31.7%**	**34.5%**	
Occasionally, sometimes	**23.5%**	**22.3%**		22.7%	22.9%		**23.7%**	**19.7%**	
Often, very often	**44.4%**	**45.2%**		44.7%	45.1%		**44.6%**	**45.7%**	
** Neighborhood can be trusted**			**P = 0.025**			P = 0.968			**P<0.001**
mean	**4.76**	**4.71**		4.74	4.72		**4.76**	**4.65**	
SE	**0.02**	**0.01**		0.01	0.02		**0.01**	**0.02**	
** Most people can be trusted**			**P<0.001**			**P<0.001**			**P<0.001**
mean	**4.97**	**4.80**		**4.91**	**4.80**		**4.91**	**4.72**	
SE	**0.02**	**0.01**		**0.01**	**0.02**		**0.01**	**0.02**	
** Get together socially with friends/relatives (%)**			**P<0.001**			**P<0.001**			**P<0.001**
Every day, several times a week	**25.4%**	**21.2%**		**24.2%**	**21.1%**		**23.6%**	**20.3%**	
About once a week, 2 or 3 times a month	**24.5%**	**28.3%**		**25.8%**	**28.2%**		**25.6%**	**31.0%**	
About once a month, once or twice every 3 months, less often than once every 3 months	**50.1%**	**50.5%**		**50.0%**	**50.7%**		**50.8%**	**48.8%**	

Numerical data are shown as mean and standard error, and categorical data are shown in proportion.

P-values were calculated by the t-test for numerical data and chi-square test for categorical data. For variables with missing values defined in the 22nd release of the HILDA data, missing values (-1 or less) were used as is, and variables with undefined missing values were treated as blanks.

**Table 2:** Relationship between socio-demographic, physical, psychological, and social factors and pet ownership (bird, fish, and others ownership).

Variable	Bird			Fish			Others		
	No (n = 14,203)	Yes (n = 1,532)	P-value	No (n = 14,532)	Yes (n = 1,203)	P-value	No (n = 14,707)	Yes (n = 1,028)	P-value
**SOCIO-DEMOGRAPHICS**									
** Sex (% female)**	**52.6%**	**57.3%**	**P<0.001**	52.9%	55.1%	P = 0.144	**52.5%**	**61.1%**	**P<0.001**
** Age**			P = 0.34			**P<0.001**			**P<0.001**
mean	46.1	45.6		**46.3**	**42.7**		**46.4**	**41.1**	
SE	0.2	0.4		**0.2**	**0.5**		**0.2**	**0.5**	
** Marital status (%)**			**P<0.001**			**P<0.001**			**P<0.001**
Legally married	**46.5%**	**54.6%**		**46.6%**	**55.5%**		**47.3%**	**47.7%**	
De facto	**16.5%**	**16.5%**		**16.6%**	**15.8%**		**16.3%**	**19.1%**	
Separated, divorced, widowed, never married and not de facto	**37.0%**	**28.9%**		**36.8%**	**28.7%**		**36.4%**	**33.2%**	
** Family member (Non-family member %)**	**17.8%**	**8.7%**	**P<0.001**	**17.6%**	**7.7%**	**P<0.001**	**17.5%**	**8.2%**	**P<0.001**
** Residential area (%)**			**P<0.001**			**P<0.001**			**P<0.001**
Major city	**63.6%**	**44.6%**		**62.3%**	**54.9%**		**63.1%**	**43.1%**	
Inner regional, outer regional, remote, very remote	**36.4%**	**55.4%**		**37.7%**	**45.1%**		**36.9%**	**56.9%**	
** Housing (%)**			**P<0.001**			**P<0.001**			P = 0.081
Owner/currently paying off home	**67.3%**	**74.3%**		**67.4%**	**74.3%**		67.7%	71.1%	
Renter (or paying board), involved in a rent-buy scheme or Live rent-free/Life Tenure	**32.7%**	**25.7%**		**32.6%**	**25.7%**		32.3%	28.9%	
** Income(A$)**			P = 0.094			**P = 0.001**			P = 0.144
mean	102,939	99,563		**102,046**	**109,433**		102,379	105,912	
SE	640	1,535		**627**	**1,900**		622	2,065	
** Annual household expenditure—per person level—fees paid to health practitioners (A$)**			P = 0.538			P = 0.572			P = 0.186
Mean	915	873		908	953		904	1014	
SE	20	89		21	67		20	131	
** Annual household expenditure–per person level—medicines, prescriptions, pharmaceuticals, alternative medicines (A$)**			P = 0.544			**P = 0.024**			P = 0.893
mean	433	447		**430**	**491**		434	430	
SE	7	24		**7**	**28**		7	36	
**PHYSICAL VARIABLES**									
** Long term impairment or disability (yes %)**	**29.1%**	**32.0%**	**P = 0.001**	29.6%	27.3%	P = 0.336	29.4%	30.2%	P = 0.870
** Limitation in activities: moderate activities (not limited at all %)**	72.7%	73.4%	P = 0.541	**72.4%**	**76.7%**	**P = 0.003**	**72.3%**	**79.2%**	**P<0.001**
** Limitation in activities: vigorous activities (not limited at all %)**	**42.2%**	**39.5%**	**P = 0.007**	41.8%	43.9%	P = 0.059	**41.6%**	**46.8%**	**P<0.001**
** Walking more than one kilometer (not limited at all %)**	74.9%	72.9%	P = 0.161	**74.4%**	**78.3%**	**P = 0.010**	**74.4%**	**79.0%**	**P = 0.009**
** Bathing or dressing (not limited at all %)**	89.3%	89.4%	P = 0.563	**89.1%**	**91.4%**	**P = 0.041**	89.1%	91.3%	P = 0.222
** Physical Activity in Wave 17**									
**Moderate physical activity (Minutes per week)**			**P<0.001**			**P = 0.041**			**P<0.001**
mean	**155.6**	**198.2**		**158.5**	**175.2**		**155.4**	**221.3**	
SE	**2.3**	**7.9**		**2.3**	**8.2**		**2.2**	**10.1**	
** Vigorous physical activity (Minutes per week)**			**P = 0.014**			P = 0.282			**P<0.001**
mean	**103.5**	**117.5**		104.3	111.2		**103.1**	**130.2**	
SE	**1.8**	**6.1**		1.8	6.4		**1.7**	**7.6**	
** Total physical activity MET (Minutes per week)**			**P<0.001**			P = 0.122			**P<0.001**
mean	**2218**	**2535**		2239	2367		**2207**	**2838**	
SE	**23**	**78**		23	84		**22**	**99**	
** Number of doctor visits in Wave 17**			**P = 0.04**			P = 0.974			P = 0.187
mean	**4.8**	**5.2**		4.9	4.9		4.9	4.6	
SE	**0.1**	**0.2**		0.1	0.2		0.1	0.2	
** Diagnosed with a serious illness in Wave 17**									
** Asthma (Yes %)**	**22.8%**	**28.9%**	**P<0.001**	**23.2%**	**26.8%**	**P = 0.043**	**22.9%**	**31.6%**	**P<0.001**
** Anxiety (Yes %)**	**22.8%**	**28.9%**	**P<0.001**	**23.5%**	**26.6%**	**P<0.001**	**23.1%**	**34.5%**	**P<0.001**
** Arthritis or osteoporosis (Yes %)**	**23.7%**	**24.5%**	**P<0.001**	**23.5%**	**26.6%**	**P<0.001**	**36.9%**	**28.5%**	**P<0.001**
** Any type of cancer (Yes %)**	**6.6%**	**5.4%**	**P<0.001**	6.5%	6.9%	P = 0.191	6.6%	4.8%	P = 0.062
** Chronic bronchitis or emphysema (Yes %)**	**4.0%**	**5.2%**	**P<0.001**	4.2%	4.1%	P = 0.201	4.2%	3.1%	P = 0.088
** Type 2 diabetes (Yes %)**	**10.5%**	**10.9%**	**P = 0.001**	10.7%	8.9%	P = 0.100	**10.8%**	**7.9%**	**P = 0.033**
** Depression (Yes %)**	**23.9%**	**26.7%**	**P<0.001**	24.0%	26.4%	P = 0.104	**23.7%**	**31.6%**	**P<0.001**
** High blood pressure or hypertension (Yes %)**	**38.4%**	**35.6%**	**P<0.001**	**38.7%**	**30.7%**	**P = 0.001**	**38.8%**	**28.8%**	**P<0.001**
** Heart disease (Yes %)**	**9.7%**	**7.9%**	**P<0.001**	**9.7%**	**6.9%**	**P = 0.028**	**9.7%**	**6.2%**	**P = 0.011**
** Other mental illness (Yes %)**	**6.2%**	**6.0%**	**P = 0.001**	5.1%	5.2%	P = 0.201	5.0%	6.4%	P = 0.083
**PSYCHOLOGICAL VARIABLES**									
** Self-assessed health (Excellent, very good %)**	**45.5%**	**40.7%**	**P = 0.007**	45.2%	43.0%	P = 0.634	45.1%	16.9%	P = 0.527
** SF-36 mental health**			P = 0.052			P = 0.492			**P = 0.019**
mean	72.3	71.4		72.3	71.9		**72.4**	**70.5**	
SE	0.2	0.5		0.2	0.5		**0.2**	**0.6**	
** Often feel very lonely**			**P = 0.090**			P = 0.877			P = 0.138
mean	**2.75**	**2.83**		2.75	2.75		2.75	2.83	
SE	**0.01**	**0.05**		0.01	0.05		0.01	0.06	
** Satisfaction with the residential home**			P = 0.259			P = 0.590			**P = 0.001**
mean	7.95	8.00		7.96	7.94		**8.10**	**7.95**	
SE	0.01	0.03		0.01	0.04		**0.01**	**0.06**	
** Feel happy as a person (%)**			**P = 0.008**			P = 0.529			P = 0.076
All of the time, most of the time	**56.7%**	**53.4%**		56.5%	55.1%		56.7%	52.1%	
A good bit of the time, some of the time, a little of the time, none of the time	**43.3%**	**46.6%**		43.5%	44.9%		43.3%	47.9%	
** Feel calm and peaceful (%)**			P = 0.307			P = 0.129			P = 0.055
All of the time, most of the time	40.6%	37.7%		40.6%	37.5%		40.6%	36.6%	
A good bit of the time, some of the time, a little of the time, none of the time	59.4%	62.3%		59.4%	62.5%		59.4%	63.4%	
** Feel I am a nervous person (%)**			P = 0.170			P = 0.405			P = 0.615
All of the time, most of the time	5.4%	5.5%		5.5%	4.8%		5.3%	6.8%	
A good bit of the time, some of the time, a little of the time, none of the time	94.6%	94.5%		94.5%	95.2%		94.7%	93.2%	
** Feel down (%)**			P = 0.142			P = 0.749			P = 0.058
All of the time, most of the time	4.8%	6.0%		4.9%	5.3%		4.8%	6.6%	
A good bit of the time, some of the time, a little of the time, none of the time	95.2%	94.0%		95.1%	94.7%		95.2%	93.4%	
** Feel full of life (%)**			**P = 0.021**			P = 0.256			P = 0.122
All of the time, most of the time	**42.6%**	**39.0%**		42.4%	41.0%		42.4%	40.1%	
A good bit of the time, some of the time, a little of the time, none of the time	**57.4%**	**61.0%**		57.6%	59.0%		57.6%	59.9%	
** Have a lot of energy (%)**			**P = 0.027**			P = 0.676			P = 0.397
All of the time, most of the time	**30.5%**	**27.4%**		30.3%	29.2%		30.3%	29.2%	
A good bit of the time, some of the time, a little of the time, none of the time	**69.5%**	**72.6%**		69.7%	70.8%		69.7%	70.8%	
**SOCIAL VARIABLES**									
** Participate in physical activity (%)**			**P = 0.017**			P = 0.480			**P = 0.005**
Not at all, less than once a week, 1 to 2 times a week or 3 times a week	**67.4%**	**67.5%**		67.3%	69.0%		**67.4%**	**67.3%**	
More than 3 times a week or Every day	**32.6%**	**32.5%**		32.7%	31.0%		**32.6%**	**32.7%**	
** Neighborhood: Neighbors help each other out (%)**			**P<0.001**			**P = 0.006**			P = 0.061
Never happens, very rare	**19.0%**	**16.2%**		**19.0%**	**15.5%**		18.9%	16.9%	
Not common, fairly common	**62.5%**	**61.7%**		**62.3%**	**64.7%**		62.6%	60.8%	
Very common	**18.4%**	**22.1%**		**18.7%**	**19.9%**		18.5%	22.3%	
** Close-knit neighborhood**			**P = 0.114**			P = 0.939			P = 0.321
mean	**3.96**	**4.02**		3.96	3.97		3.96	4.01	
SE	**0.01**	**0.04**		0.01	0.04		0.01	0.05	
** Chat with neighbors (%)**			P = 0.269			**P = 0.003**			**P = 0.034**
Never, rarely	32.7%	29.8%		**32.6%**	**29.7%**		**32.2%**	**34.6%**	
Occasionally, sometimes	44.7%	46.3%		**44.8%**	**46.0%**		**45.0%**	**43.3%**	
Often, very often	22.6%	23.9%		**22.6%**	**24.3%**		**22.8%**	**22.1%**	
** Neighborhood can be trusted**			P = 0.674			P = 0.798			P = 0.929
mean	4.73	4.72		4.73	4.74		4.73	4.73	
SE	0.01	0.04		0.01	0.04		0.01	0.04	
** Most people can be trusted**			**P = 0.002**			P = 0.360			**P = 0.005**
mean	**4.87**	**4.76**		4.87	4.83		**4.87**	**4.75**	
SE	**0.01**	**0.03**		0.01	0.04		**0.01**	**0.04**	
** Get together socially with friends/relatives (%)**			**P<0.001**			**P = 0.040**			**P<0.001**
Every day, several times a week	**23.3%**	**19.1%**		**23.1%**	**20.3%**		**23.1%**	**19.3%**	
About once a week, 2 or 3 times a month	**50.8%**	**46.2%**		**50.4%**	**49.7%**		**50.6%**	**46.5%**	
About once a month, once or twice every 3 months, less often than once every 3 months	**26.0%**	**34.7%**		**26.6%**	**30.0%**		**26.3%**	**34.2%**	

Numerical data are shown as mean and standard error, and categorical data are shown in proportion.

P-values were calculated by the t-test for numerical data and chi-square test for categorical data. For variables with missing values defined in the 22nd release of the HILDA data, missing values (-1 or less) were used as is, and variables with undefined missing values were treated as blanks.

### 3.3. Main analyses for the associations of pet ownerships with all-cause mortality

During the 4-year follow-up period, 377 of 15,735 (2.4%) participants died. The mean age of participants who died was 75.1 (14.8) years. By pet ownership, 148 of 9,525 (1.6%) pet owners and 229 of 6,210 (3.7%) non-pet owners died during the follow-up period. The proportion of deaths was 106 (1.5%) for dog owners, 63 (1.7%) for cat owners, 25 (1.6%) for bird owners, 13 (1.1%) for fish owners, and 12 (1.2%) for other owners.

Results for the inverse probability of treatment weighted logistic regression model with propensity score showed that pet owners had ORs (95% CI: Confidence Interval) of 0.74 (95%CI: 0.59–0.93) for all-cause mortality compared with non-pet owners in Model 2 (Table 3). Compared with non-pet owners, ORs for all-cause mortality were 0.77 (95%CI: 0.59–0.99) for dog owners. Ownership of cats, birds, fish, and others showed lower ORs compared with non-pet owners, but no significant association with mortality in any case. Sensitivity analysis which excluded deaths during the first year of the follow-up period showed that dog owners had ORs of 0.74 (95%CI: 0.55–1.00) for all-cause mortality, compared with non-pet owners in Model 2 (S1 Table). Model 2 has a 3.5% smaller sample size than the crude model due to missing values.

**Table 3:** Associations between pet ownership (dog, cat, bird, fish, and others) and all-cause mortality.

	Any pet	Dog	Cat	Bird	Fish	Other
Crude OR for owners (95%CI) ^※^^1^	**0.41 (0.33–0.51)** **P<0.001**	**0.41 (0.32–0.52)** **P<0.001**	**0.45 (0.34–0.60)** **P<0.001**	**0.43 (0.29–0.66)** **P<0.001**	**0.29 (0.16–0.50)** **P<0.001**	**0.45 (0.17–0.55)** **P<0.001**
Model 1 OR for owners (95%CI) ^※^^2^	**0.72 (0.57–0.90)** **P = 0.004**	**0.74 (0.57–0.95)** **P = 0.021**	0.75(0.54–1.02) P = 0.067	0.68(0.39–1.18) P = 0.168	0.68 (0.30–1.54) P = 0.346	0.87 (0.36–2.08) P = 0.750
Model 2 OR for owners (95%CI) ^※^^3^	**0.74 (0.59–0.93)** **P = 0.010**	**0.77 (0.59–0.99)** **P = 0.044**	0.77 (0.56–1.05) P = 0.105	0.69 (0.40–1.22) P = 0.205	0.68 (0.30–1.54) P = 0.335	0.80 (0.33–1.92) P = 0.619

OR, odds ratio; CI, confidence interval. Reference group were non-pet owners.

※1 n = 15,735, ※2 n = 15,735, ※3 n = 15,172.

Analysis was weighted by the inverse of propensity score in the GEE (Generalized estimating equation). Model 1: weight was calculated based on sex, age, marital status, family members, housing, and income. Model 2: weight was calculated Model 1 plus limitation in vigorous activities, walking more than one kilometer, self-assessed health, SF-36 mental health, neighbors help each other out, get together socially with friends/relatives, any type of cancer, type 2 diabetes, and high blood pressure or hypertension.

### 3.4. Supplementary analysis: Mediation analysis and average treatment effects

The Sobel test showed a partial mediating effect of physical activity level (total physical activity MET) on the relationship between dog ownership and all-cause mortality. The indirect effect was -0.001 (z = -5.250, p<0.001), and the ratio of the indirect effect to total effect (RIT) was 7%.

## 4. Discussion

This nationally representative prospective study revealed that dog owners had low OR for mortality compared to non-pet owners after controlling for related socio-demographic, physical, psychological, and social factors during a 4-year follow-up period. The present study showed that physical activity level had a partial mediating effect on the relationship between dog ownership and mortality.

In 1980, Friedmann et al. reported that post-myocardial infarction or angina pectoris patients with pet ownership had a higher 1-year survival rate than non-pet owners [10], and similar results in a subsequent study in 1995 [17]. In the decade of the 90s, epidemiological studies focused on the types of pets and examined the association of dog and/or cat ownership with mortality [9, 11–14, 16, 20]. Most previous studies, including a systematic review and meta-analysis [14], concluded that dog ownership had a positive effect on mortality among middle-aged [11–13, 16] and older adults [9, 11, 16] in the US [16], UK [13], Sweden [11, 12], and Japan [9]. In our present study, compared to non-pet owners, dog owners had an OR of 0.77 (95%CI: 0.59–0.99) for mortality after controlling for a wide range of socio-demographic, physical, psychological, and social factors during a 4-year follow-up period in Australia. This result that dog ownership has a positive association with mortality is consistent with previous evidence.

In this study, we also examined the underlying mechanism between dog ownership and mortality. A plausible benefit of dog ownership is its effect in maintaining and increasing physical activity level [2, 33, 34]. We previously reported that dog owners with a daily exercise habit had significantly low odds ratios for the onset of dementia [8] and disability [9]. Physical activity level is a well-known risk factor for mortality [35]. Our present study shows a partial mediating effect of physical activity level on the relationship between dog ownership and mortality. Kramer et al. suggested that the lower risk of death with dog ownership is possibly driven by a reduction in cardiovascular mortality [14]. Although our data in this study did not include cause of death, we suggest that dog ownership has protective effects on cardiovascular mortality through a higher physical activity level. Further, we found that ownership of cats, birds, fish, and other animals had no clear association with mortality. Gillum et al. reported that living with a cat or other animal was not associated with lower risk of death independent of confounders compared to those with no companion animal [20]. Indeed, several studies have shown negative associations with mortality: women with a cat had higher risk of lung cancer mortality than women with no pet [18]; cat owners had higher risk of colorectal cancer mortality than non-pet owners [15]; and cat and bird owners had higher risk of cancer mortality, particularly women, than non-pet owners [19]. Conversely, Ogechi et al. reported that cat owners rather than dog owners had lower risk of cardiovascular mortality, in particular death by stroke [16]. Considering this previous inconsistent evidence, cat and bird ownership might have different associations with either sex or specific causes of death. Our present nationally representative study showed that cat and bird ownership did not have any impact for mortality, which overlaps with previous evidence for cat [20] and bird ownership [15, 18], respectively. To our knowledge, this study is the first to examine the association of fish and other animal ownership (including horses, rabbits, guinea pigs/hamsters, lizards, snakes, turtles/tortoises, sheep, cows, goats, rats/mice, alpacas, and others) with mortality. In this study, fish and other animal owners showed relatively low crude odds ratio for mortality (ORs = 0.68 and 0.80, respectively); after controlling for a wide range of socio-demographic, physical, psychological, and social factors, however, no clear associations were found. A few experimental studies have examined physiological stress on watching the videotape of an aquarium [20, 36]. In future studies, it will be necessary to follow fish and other animal owners for a longer term and examine the association of their ownership with health outcomes.

This study has several main strengths. First, the HILDA survey–the data source for this study–is a large-scale, nationally representative household panel survey in Australia. The HILDA data are derived from 488 CDs and with various residential areas and states, enabling us to identify related socio-demographic, physical, psychological, and social factors when the number of pet owners was small, such as for fish, and others. Second, a wide range of variables were used to calculate weighting, and we were able to perform an inverse probability of treatment weighted logistic regression model with socio-demographic, physical, psychological, and social variables. The characteristics of dog and cat owners in this study, such as housing [37] and physical function [33], were consistent with previous studies. The present study used a maximum of 15 socio-demographic, physical, psychological, and social variables to examine association of pet ownership with mortality.

This study also has some limitations. First, the 4-year follow-up period is relatively short compared with previous studies. Future studies should extend the follow-up period and examine the association of pet ownership with risk of all-cause mortality. To discuss the causality of pet ownership with mortality in this study, we conducted a sensitivity analysis which excluded deaths occurring in the first year of follow-up. Second, although we discuss cause of death as cardiovascular death among dog owners and non-dog owners, data on cause of death in this study are not available at present. A future study is required to examine the association of dog ownership with cardiovascular mortality. Third, several important variables could not be included in this study. Attachment to a pet is known to affect psychological aspects [38, 39], and primary caretaking with a deep owner-pet relationship might have a key role in this effect on human health. Fourth, data on physical activity level and serious illness in this study were collected 1 year before pet ownership. A future study should assess these important variables at the same time as pet ownership to clarify the mechanism of why non-dog owners had higher mortality risk. Lastly, this study examined the association but not the causal effects between pet ownership and mortality.

## 5. Conclusion

This nationally representative prospective study revealed that dog owners had an OR of 0.77 (95%CI: 0.59–0.99) for mortality compared to non-pet owners after controlling for related socio-demographic, physical, psychological, and social factors during a 4-year follow-up period in Australia. The present study showed that physical activity level had a partial mediating effect on the relationship between dog ownership and mortality. In contrast, ownership of cats, birds, fish, and other animals had no clear association with mortality in this study. Companionship and exercise of a pet dog may be recommended as a component of health promotion policy, and may have an important role to play in promoting health aging.

## Supporting information

S1 Table Sensitive analysis for associations between pet ownership (dog, cat, bird, fish, and others) and all-cause mortality excluding data from the first year of the follow-up period. (DOCX)

S1 Appendix (DOCX)
