# Supplementary material for: Dog, cat, bird, fish, and other pet ownership and mortality: Evidence from the HILDA cohort
Source: PLoS One. 2024 Aug 14;19(8):e0305546. doi: 10.1371/journal.pone.0305546 (PMC11324118; doi:10.1371/journal.pone.0305546)
Supplement: S1 Table — (DOCX) [file pone.0305546.s001.docx]

| Supplemental Table 1. Sensitive analysis for associations between pet ownership (dog, cat, bird, fish, and others) and all-cause mortality excluding data from the first year of the follow-up period. | | |
| --- | --- | --- |
|  | Any pet | Dog |
| Crude OR for owners (95%CI) ^※1^ | **0.46 (0.36-0.59)**  **P<0.001** | **0.43 (0.33-0.57)**  **P<0.001** |
| Model 1 OR for owners (95%CI) ^※2^ | 0.83 (0.64-1.08)  P=0.168 | **0.73 (0.54-0.98)**  **P=0.036** |
| Model 2 OR for owners (95%CI) ^※3^ | 0.81 (0.62-1.04)  P=0.105 | **0.74 (0.55-1.00)**  **P=0.047** |
| OR, odds ratio; CI, confidence interval. Reference group were non-pet owners. ※1 n=15,735, ※2 n=15,735, ※3 n=15,172.  Analysis was weighted by the inverse of propensity score in the GEE (Generalized estimating equation). Model 1: weight was calculated based on sex, age, marital status, number of family members, housing, and income. Model 2: weight was calculated from Model 1 plus limitation in vigorous activities, walking more than one-kilometer, self-assessed health, SF-36 mental health, neighbors help each other out, get together socially with friends/relatives, any type of cancer, type 2 diabetes, and high blood pressure or hypertension. | | |
