# Supplementary material for: Dog, cat, bird, fish, and other pet ownership and mortality: Evidence from the HILDA cohort
Source: PLoS One. 2024 Aug 14;19(8):e0305546. doi: 10.1371/journal.pone.0305546 (PMC11324118; doi:10.1371/journal.pone.0305546)
Supplement: S1 Appendix — (DOCX) [file pone.0305546.s002.docx]

**Appendix**

***Description of variables***

Income was calculated as follows: the sum across all household members of financial year gross regular income minus taxes on financial year gross regular income [28, 29]. Per-person fees paid to health practitioners and per-person payments for medicines, prescriptions, pharmaceuticals, and alternative medicine were calculated by the amount averaged across individuals providing responses.

Long term impairment or disability was enquired about as follows: Do you have any long-term health condition, impairment or disability (such as the following) that restricts you in your everyday activities, and has lasted or is likely to last for 6 months or more? Limitation in moderate activities was enquired about as follows: Does your health now limit you in activities such as moving a table, pushing a vacuum cleaner, bowling or playing golf? Limitation in vigorous activities was enquired about as follows: Does your health now limit you in activities such as running, lifting heavy objects, participating in strenuous sports? Physical activity levels were assessed by the short-form of the IPAQ [30].

SF-36 mental health [31] was calculated as follows: items were recoded as required, raw scale scores were calculated by summing across the items in the same scale; and these raw scores were transformed to a 0-100 scale. In accordance with the manual, a person-specific raw score was estimated for any scale on which there were valid responses for equal to or greater than half the items, the average being calculated and applied to missing data. Often feel very lonely was scored from 1 (strongly disagree) to 7 (strongly agree). Satisfaction with the residential home was scored from 0 (totally dissatisfied) to 10 (totally satisfied). Feeling of happiness as a person, feeling of calm and peacefulness, feeling of being a nervous person, feeling down, feeling full of life, and having a lot of energy were scored from 1 (all of the time) to 6 (none of the time), respectively.

Enquiries on whether the participant lived in a close-knit neighborhood, the neighborhood can be trusted, and whether most people can be trusted were scored from 1 (strongly disagree) to 7 (strongly agree).
